# Intelligent wireless walls for contactless in-home monitoring

**DOI:** 10.1038/s41377-022-00906-5

**Published:** 2022-07-07

**Authors:** Muhammad Usman, James Rains, Tie Jun Cui, Muhammad Zakir Khan, Jalil ur Rehman Kazim, Muhammad Ali Imran, Qammer H. Abbasi

**Affiliations:** 1 University of Glasgow, James Watt School of Engineering, Glasgow, G12 8QQ UK; 2 State Key Laboratory of Millimetre Waves, Southeast University, Nanjing, China

**Keywords:** Microwave photonics, Metamaterials

## Abstract

Human activity monitoring is an exciting research area to assist independent living among disabled and elderly population. Various techniques have been proposed to recognise human activities, such as exploiting sensors, cameras, wearables, and contactless microwave sensing. Among these, the microwave sensing has recently gained significant attention due to its merit to solve the privacy concerns of cameras and discomfort caused by wearables. However, the existing microwave sensing techniques have a basic disadvantage of requiring controlled and ideal settings for high-accuracy activity detections, which restricts its wide adoptions in non-line-of-sight (Non-LOS) environments. Here, we propose a concept of intelligent wireless walls (IWW) to ensure high-precision activity monitoring in complex environments wherein the conventional microwave sensing is invalid. The IWW is composed of a reconfigurable intelligent surface (RIS) that can perform beam steering and beamforming, and machine learning algorithms that can automatically detect the human activities with high accuracy. Two complex environments are considered: one is a corridor junction scenario with transmitter and receiver in separate corridor sections and the other is a multi-floor scenario wherein the transmitter and receiver are placed on two different floors of a building. In each of the aforementioned environments, three distinct body movements are considered namely, sitting, standing, and walking. Two subjects, one male and one female perform these activities in both environments. It is demonstrated that IWW provide a maximum detection gain of 28% in multi-floor scenario and 25% in corridor junction scenario as compared to traditional microwave sensing without RIS.

## Abstract (web summary)

This work proposes a concept of intelligent wireless walls (IWW) to ensure high-precision activity monitoring in complex environments, namely corridor junction scenario and multi-floor scenario

## Graphical abstract

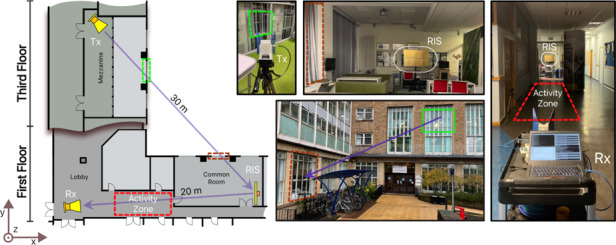

## Introduction

Human activity and motion detection have gained significant attraction from research community for their applications in remote healthcare monitoring, intrusion detection and independent living. Indeed, independent living is included in the national agenda of the UK for 2030 under the policy of good health and sustainable communities^1^. Various human activity recognition systems have been proposed in the literature exploiting ambient sensors, cameras, and wearables. However, these techniques raise either privacy concerns or discomfort of carrying wearables all the time. These concerns can be addressed by exploiting a contact-less human activity monitoring system. In this regard, various contact-less solutions are proposed in the literature, exploiting channel state information (CSI) of microwave-based wireless systems, such as WiFi^2^ and 5G^3^ or Doppler signatures of radar systems^4^. The principle of microwave sensing is based on observing the variations in the reflected signal due to movement of the target. Useful information about the target can be derived by processing the reflected signals.

However, current microwave-based activity monitoring systems have some fundamental disadvantages that limit their applicability in real-life environments. First, because the target’s reflection signals are weaker than line-of-sight (LOS) signals, the detection range is restricted to a few metres only^5^. Secondly, for contactless activity monitoring, interference from the environment is a problem. When the target’s reflection signal and an interfering reflection signal are combined at the receiver, the sensing performance is dramatically reduced. This limits the use of microwave sensing in non-LOS scenarios where the transmitter (Tx) and the receiver (Rx) do not have a direct wireless link. Above all, most microwave sensing schemes require controlled ideal settings where the movements are pseudo-dynamic.

These limitations can be overcome by beamforming towards the target to enhance the sensing range and avoid interference^6^. To this aim, a concept of intelligent wireless walls (IWW) is presented, which is based on reconfigurable intelligent surfaces (RISs) and machine learning algorithms to detect human activities with high resolution. RISs are electromagnetic (EM) metasurfaces whose electrical and optical properties (i.e. surface-averaged susceptibility) are dynamically controlled, allowing incident EM waves to be steered in the desired direction^7,8^. IWW push the limits of microwave sensing by actively steering the ambient microwave signals towards a specific area in space, enabling high-precision sensing and activity monitoring.

Recent advances in artificial intelligence (AI) and composite materials have given rise to intelligent communication and imaging systems based on RIS. RISs are typically composed of two layers, the first layer being a metasurface structure composed of tunable, subwavelength unit cell elements, usually metallodielectric in nature, with subwavelength unit cell spacing, with a second layer accommodating a control and biasing network. The metasurface layer is of subwavelength thickness, is transversally electrically large, and is globally passive in nature. Compared to technologies such as phased arrays and relays, a major advantage of employing RISs for EM transformations is their low complexity and passive nature, thereby making the technology easily scalable to cover large surface areas at low manufacturing cost, and with minimal power consumption. With many elements to configure comes the challenge of selecting an RIS configuration from a vast number of possibilities to realise a desired set of EM transformations.

The use of RIS for high-precision sensing has recently gained attention. Li et al.^9^ have demonstrated the use of RIS in recognising the objects and gestures in the surroundings with low latency. The authors introduced the concept of learned sensing in recognising gestures and constructing microwave imaging. Similarly, a machine learning-enabled RIS is proposed in ref. ^10^ as electronically controlled metasurface imager. Further, a smart metasurface imager and recogniser in conjunction with a network of artificial neural networks to manage data flow in an adaptive manner is proposed^11^. The authors propose an intelligent interface between humans and devices that allows gadgets to detect and recognise more complex human actions.

A worth mentioning related research area here is the imaging and/or tracking of objects in non-line-of-sight (Non-LOS) environments. Various different technologies have been utilised in the literature to achieve Non-LOS imaging. For instance, the authors in ref. ^12^ propose a Non-LOS acoustic imaging for the corner objects. A pair of speaker and microphone is utilised for sound waves emission and recording after reflection. However, to reconstruct a 3D image reflection, measurements are captured from a range speaker and microphone position, which limits its wide adoption in real-time applications. Likewise, a long-wave infra-red (IR) based Non-LOS imaging framework is presented in ref. ^13^. The authors demonstrated 2D shape reconstruction of hidden object to estimate the pose. Further, the work presented in ref. ^14^ achieve tracking occluded objects outside direct LOS using a standard 2D camera and a laser pointer. However, these approaches^13,14^ require recording objects using either an IR camera^13^ or a standard 2D camera^14^, which may raise privacy issues for some users. Similarly, a passive sensing approach^15^ using spatial coherence of the reflected light from a defusing wall can be used to retrieve geometric information of objects hidden in the Non-LOS locations. Nevertheless, the practicality of such an approach^15^ can be challenged in poor or no lightening. Furthermore, a radar-based Non-LOS target detection and localisation is presented^16^, where reflections and/or diffractions on the surrounding surfaces are utilised along with the scene geometry.

Empowered with AI, this work aims to offer a paradigm shift in contactless in-home activity monitoring by introducing an RIS to extend the coverage region of an activity monitoring system. The sensing capabilities of the RIS-aided activity monitoring system are then demonstrated in complex wireless propagation environments where conventional microwave sensing does not perform well. Namely, sensing around a corner in a corridor junction and sensing across multiple floors.

A related work to see around the corner using RIS is presented by ref. ^17^. The authors^17^ considered the use of RISs to extend radar surveillance to NLoS scenarios. The work derived a system model for monostatic radar aided by a RIS. The authors determined an approximation for the SNR and expressions for signal-to-clutter ratio (SCR) accounting for surface or volume clutter. Numerical analysis involving the detection of a micro unmanned aerial vehicle revealed, for an ideal RIS with full phase tuneability, produced a significant improvement in SNR and SCR with increased RIS size. While Aubry et al. consider a numerical example of the monostatic case, we can consider our experimental contribution as a bistatic case. The authors consider conventional radar signal processing in their formulations, whereas we consider the range extension of an ambient sensing-based activity monitoring system where the transmitter and receiver are located at opposing sides of a blockage.

To manipulate the wireless channel, we have utilised in this work an RIS testbed with high resolution beamsteering capability in the azimuthal plane. This device was recently shown to offer significant indoor coverage enhancement performance when deployed in Non-LOS communication scenarios^18^. This RIS consists of many connected columns of sub-wavelength unit cells, with a near-3 bit phase resolution provided by integration of 3 PIN diodes within each unit cell. The RIS is controllable remotely over a WiFi link, such that it can be easily integrated into existing network infrastructure.

In this work, a novel concept of IWW is presented wherein the limitations of Non-LOS microwave sensing are overcome by utilising RIS and AI. Two complex environments are considered wherein transmitter and receiver are either in separate corridors or placed on different floors of a building. In each scenario, three different body postures are considered, namely sitting, standing, and walking. Different machine learning algorithms are investigated to correctly classify the considered body postures in RIS and without RIS settings. To the best of authors’ knowledge, RIS has not been considered to monitor activities of a target elsewhere in the literature in corridor junction and multi-floor scenarios. Moreover, the ultimate goal of this study is to investigate the capabilities of RIS in extending the useful range of real-time activity monitoring systems for facilitating independent living.

## Results

This section highlights the performance of considered machine learning algorithms in both scenarios (corridor junction and multi-floor) with and without RIS. The performance of both evaluation techniques (RS) *k*-fold cross validations and train-test split is presented in this section.

### Corridor junction scenario

First of all, the evaluation of three machine learning algorithm, i.e. Random Forest (RF), Extra Trees (ET) and Multilayer Perceptrons (MLP) in corridor junction scenario is presented in Tables 1 and 2. In particular, Table 1 depicts the results of test-train evaluation technique. It can be observed from the table that the accuracy of individual participants (i.e. S1 and S2) reaches 100% while RIS is on and Tx and Rx are forming a virtual communication link via RIS. Turning on RIS gives 25% accuracy gain with MLP algorithm on S2, which gives only 75% accuracy while RIS is off. The combined accuracy of both participants (S1+S2) is 100% using RF algorithm when RIS is on, while the classification accuracy of this algorithm is only 75% when RIS is turned off.

**Table 1:** Classification accuracy of machine learning algorithms in corridor junction scenario using test-train evaluation method.

S.No	Algorithms	RIS-off (S1)	RIS-on (S1)	RIS-off (S2)	RIS-on (S2)	RIS-off (S1+S2)	RIS-on (S1+S2)
1	RF	93.75	100	93.75	100	75.00	100
2	ET	93.75	100	95.47	100	71.87	96.87
3	MLP	93.75	100	75.00	100	75.00	87.50

**Table 2:** Classification accuracy of machine learning algorithms in corridor junction scenario using repeated stratified *k*-fold validation.

S.No	Algorithms	RIS-off (S1)	RIS-on (S1)	RIS-off (S2)	RIS-on (S2)	RIS-off (S1+S2)	RIS-on (S1+S2)
1	RF	85.66	100	91.10	100	81.80	89.58
2	ET	90.81	100	94.27	100	83.53	91.47
3	MLP	80.23	99.58	86.01	97.38	75.04	89.95

Similarly, Table 2 presents the classification accuracy of the same machine learning algorithms in corridor junction scenario considering (RS) *k*-fold cross-validation. Again here, RIS gives significant performance improvement over conventional microwave sensing (RIS-off). The accuracy of individual participants is 100% for RF and ET algorithms, while combined dataset generates 91.47% classification accuracy using ET algorithm. The maximum accuracy gain in the cross validation method over conventional microwave sensing (RIS-off) is around 20% on S1 dataset using MLP algorithm.

**Fig. 1:**
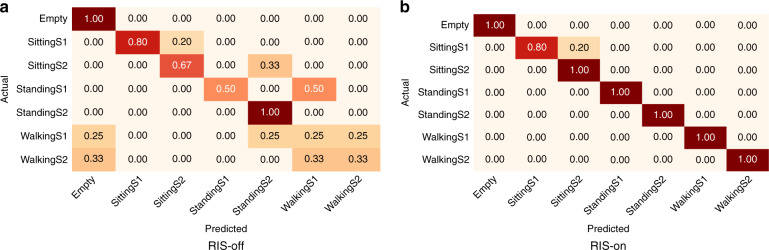
Normalised confusion matrix of combined data samples in corridor junction scenario using train test evaluation method. **a** RIS-off scenario, where many classes are wrongly classified. **b** RIS-on scenario, where majority of the classes are correctly classified.

Further, Fig. 1 illustrates the confusion matrix of ET algorithm in classifying all seven classes, i.e. sitting, standing, and walking of both participants and empty. Figure 1a represents the normalised confusion matrix while RIS is off and Fig. 1b represents the normalised confusion matrix while RIS is on. It can be noted from the confusion matrix that while RIS is off only a few classes are rightly classified. Further, walking activities of both participants are mostly wrongly classified. On the other hand, once Tx and Rx create a virtual LOS link via RIS, the classification accuracy increases for all classes (Fig. 1a). The maximum wrongly classified accuracy is for sitting activity of S1, which has only 20% incorrect classification. Rest all classes shown 100% classification accuracy with RIS-on.

### Multi-floor scenario

The evaluation results of the same three machine learning algorithms in multi-floor scenario are presented in Tables 3 and 4. While Table 3 depicts the classification accuracy of test-train evaluation method, Table 4 does the same for (RS) *k*-fold cross validation technique. It can be noted from both tables that while RIS is off, i.e. there is no LOS link between Tx and Rx, the classification accuracy is poor for individual participants and on the combined dataset. For instance, the classification of as low as 51.42% is observed on combined dataset with RF algorithm and test-train evaluation model. While an accuracy of only 51.38% is observed for the same algorithm on the same dataset with (RS) *k*-fold cross validation model. When optimised, the RIS significantly increases the classification accuracy in all cases. For instance, the maximum classification accuracy of 86.52% is observed on S2 dataset with an accuracy gain of more than 11%. This verifies the idea of detection resolution increase with RIS in both cases.

**Table 3:** Classification accuracy of machine learning algorithms in multi-floor scenario using test-train evaluation method.

S.No	Algorithms	RIS-off (S1)	RIS-on (S1)	RIS-off (S2)	RIS-on (S2)	RIS-off (S1+S2)	RIS-on (S1+S2)
1	RF	63.00	82.50	70.00	79.5	51.42	72.14
2	ET	70	92.50	80.00	85.00	54.28	81.42
3	MLP	62.50	57.50	77.50	91.66	52.85	54.28

**Table 4:** Classification accuracy of machine learning algorithms in multi-floor scenario using repeated stratified *k*-fold validation.

S.No	Algorithms	RIS-off (S1)	RIS-on (S1)	RIS-off (S2)	RIS-on (S2)	RIS-off (S1+S2)	RIS-on (S1+S2)
1	RF	57.79	78.51	73.74	84.86	51.38	69.25
2	ET	64.01	84.28	75.39	86.52	55.67	85.71
3	MLP	62.63	65.54	73.89	79.92	52.25	49.59

**Fig. 2:**
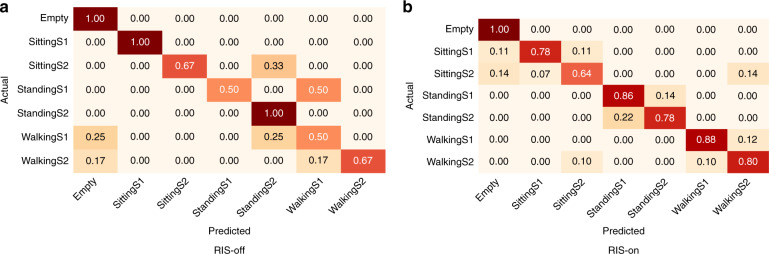
Normalised confusion matrix of combined data samples in multi-floor scenario using train test evaluation method. **a** RIS-off scenario, where many classes are wrongly classified. **b** RIS-on scenario, where majority of the classes are correctly classified.

Similarly, Fig. 2 illustrates confusion matrix of ET algorithm in classifying the same seven classes. Figure 2a represents the normalised confusion matrix while RIS is off and Fig. 2b represents the normalised confusion matrix while RIS is on. It can be noted from the confusion matrix that while RIS is off only few classes are rightly classified. Further, walking and standing activities of S2 are mostly wrongly classified. On the other hand, once RIS is turned on, the classification accuracy increases for all classes (Fig. 2b). In this case, the maximum wrongly classified accuracy is for sitting activity of S2, which has 26% incorrect classification.

**Fig. 3:**
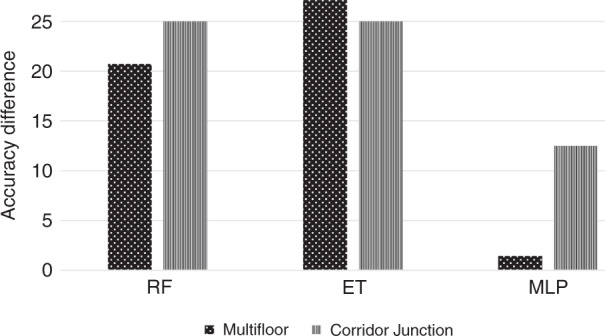
The accuracy difference of machine learning algorithms between RIS-off and RIS-on in both scenarios. The value of each bar in the figure is calculated by subtracting the classification accuracy of RIS-off from the accuracy of RIS-on.

Figure 3 represents the accuracy difference among all considered algorithms on both multi-floor and corridor junction scenarios with RIS-off and RIS-on. The difference is calculated on the combined dataset. The difference is calculated by subtracting the accuracy of RIS-off from the accuracy of RIS-on. It can be noted that the values of all bars are positive, which verifies that the RIS always increases the detection accuracy in both considered environments, i.e. corridor junction and multi-floor. The maximum difference is for ET algorithm in multi-floor scenario, which shows that exploiting RIS in multi-floor scenario increases the classification accuracy by more than 25%. This accuracy increase is due to the beamforming capabilities of RIS, which results in getting more reflection from the body of the participant and increases the detection accuracy.

## Discussion

**Fig. 4:**
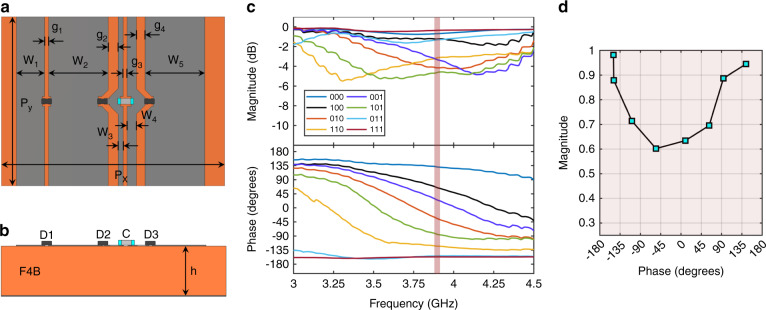
Unit cell design utilised in this work. Front view (**a**) and cut view (**b**) with associated dimensions. The measured reflection response versus frequency for the eight PIN diode configurations is shown in **c**. The phase versus magnitude at 3.9 GHz is shown in **d**, highlighting the non-negligible phase-dependent magnitude response of the RIS.

The RIS utilised in this work is based on the unit cell recently published by ref. ^19^ and is depicted in Fig. 4. The unit cell dimensions can be found in Table 5. The unit cells consist of five copper patches connected by three PIN diodes and a capacitor. The patches are etched onto a grounded F4BM-2 dielectric substrate with relative permittivity *ϵ* = 2.65 and loss tangent tan*δ* = 0.001. Referring to Fig. 4, the patches are connected to neighbouring unit cells at the top and bottom in order to reduce the configuration network complexity at the cost of reflection control in elevation.

**Table 5:** Dimensions for multi-bit unit cell design.

Parameter	Periodicity, *P*_*x*_*P*_*y*_	Patch width, *W*_1_ to *W*_5_	Patch spacing, *g*_1_ to *g*_4_	Substrate thickness, *h*
Dimension (mm)	22.5, 15.0	6.0, 0.9, 0.5, 6.0, 2.9	0.9, 0.4, 1.0, 0.4	5.0

The local reflection response for each unit cell can be changed by varying the respective PIN diode biasing states. Between adjacent patches, a forward-biased PIN diode acts as a small series resistance, whereas a reverse-biased PIN diode acts as a series capacitor. With the three PIN diodes, eight biasing combinations are available, denoted as binary values 000 to 111 in Fig. 4d. The reverse-biased state in this design is achieved as a 0V control voltage, whereas the forward-biased state is ~0.85 V with a 3 mA forward current. The capacitor, operating at its self-resonant frequency, provides a near-short circuit at the operating frequency of 3.75 GHz whilst isolating the DC bias signal paths. The patch dimensions were optimised to maximise the phase resolution of the set of reflection responses. For this design, seven distinct reflection phase states are available, with two of the eight biasing combinations exhibiting a similar response (i.e. 011 and 111).

**Fig. 5:**
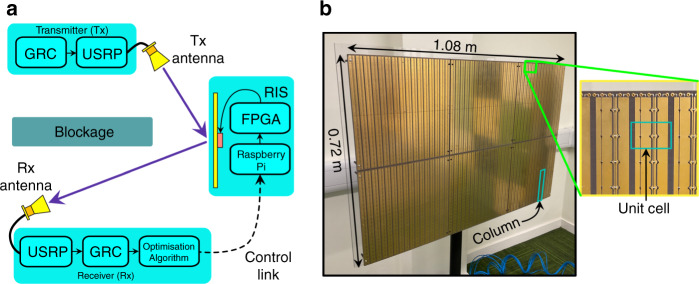
RIS-aided single-input single-output (SISO) wireless communication system. **a** The RIS and receiver (Rx) form a feedback loop to iteratively increase the channel gain from the transmitter (Tx) to the Rx antenna, circumventing the blockage. **b** Fabricated reconfigurable intelligent surface. Inset shows column structure with choke inductors at the top. Column highlighted consists of a group of 12 unit cells whose patches are connected top and bottom.

The fabricated prototype is shown in Fig. 5b and consists of an arrangement of 48 × 48 unit cell elements, connected in columns of 12 (i.e. 4 rows, 48 columns long). The RIS dimensions are 1.08 m in width and 0.72 m in height or 13.5*λ* × 9*λ* at 3.75 GHz. The PIN diodes on the unit cells receive bias voltages from a network of shift registers on the back of the RIS. An RIS control link is facilitated by a WiFi link to a Raspberry Pi single-board computer. Configurations from a PC are sent over a socket connection, converted into a binary stream, and parallelised to the RIS shift registers via an FPGA. Alternatively, to reduce configuration time, RIS configurations can be pre-loaded into the FPGA’s flash memory.

### RIS configuration algorithm

The physical behaviour of an RIS can be explained by Huygen’s principle^20^. In response to an incident EM wave, each unit cell element can be treated as a source of a spherical wave, whose phase relationship to adjacent sources is determined by the reradiation properties of the RIS unit cells. The received power at a point in space, as thoroughly outlined by ref. ^21^, is proportional to the superposition of the reradiated wave components from the set of *N* × *M* unit cells each with lateral dimensions *d*_*x*_ by *d*_*y*_, satisfying the relationship:

1 $${P}_{r}\propto {P}_{t}\frac{{G}_{t}{G}_{r}{d}_{x}{d}_{y}{\lambda }^{2}}{64{\pi }^{3}}{\left|\mathop{\sum }\limits_{m = 1-\frac{M}{2}}^{M/2}\mathop{\sum }\limits_{n = 1-\frac{N}{2}}^{N/2}\frac{{{{\Gamma }}}_{n,m}}{{r}_{n,m}^{t}{r}_{n,m}^{r}}\exp \left(-j\frac{2\pi }{\lambda }[{r}_{n,m}^{t}+{r}_{n,m}^{r}]\right)\right|}^{2}$$

with *G*_*r*_, *G*_*t*_, *P*_*t*_, and *λ* the Rx antenna gain, Tx antenna gain, transmit power, and free-space wavelength, respectively. ${r}_{n,m}^{t}$ and ${r}_{n,m}^{r}$ are the respective distances between unit cell (*n*,*m*) and the Tx and Rx antennas. The local reflection coefficients, ${{{\Gamma }}}_{n,m}\in [{\rho }_{1}{e}^{j{\phi }_{1}},...,{\rho }_{8}{e}^{j{\phi }_{8}}]$, account for the complex reflection responses of the constituent unit cells, each of which if set to one of the eight available biasing states. Equation (1) should contain a term accounting for the Tx and Rx antenna beam patterns, as well as the unit cell reception and reradiation patterns proportional to the unit cell area^21^.

To maximise the power intercepted by the Rx, it can be seen from eq. (1) that the phases of the many paths via the set of RIS unit cells should add coherently at the Rx antenna. However, due to the phase-dependent magnitude of the unit cell reflection behaviour, it may not always be the optimal choice to select the reflection states which provide this phase coherence alone^22^. In this work, we employ an adaptive optics-based RIS optimisation algorithm in a similar fashion to ref. ^23^. During the optimisation stage, all unit cell are initially set to an unbiased state. The bias states of the top-left column of the RIS are iterated through and the power at Rx is sampled for each one. This is followed by setting the first column grouping (i.e. the first grouping of 12 unit cells in the top row) to the state which resulted in the highest received power. This is repeated for the remaining columns, holding the subsequent columns fixed at the states which resulted in the highest power. Although very basic and likely much more computationally expensive than many of the proposed RIS optimisation techniques^24^, this algorithm is easy to employ and is guaranteed to converge^25^.

## Materials and methods

This section explains the considered experimental scenarios and the hardware and software details of the complete experimental setup used in these scenarios.

### Experiment setup

Two Non-LOS environments are considered where conventional microwave sensing does not perform well. One is at a right-angled corridor junction, where Tx and Rx do not have any direct communication link. The second scenario involved propagation across multiple floors, where the Tx was placed on the third floor of an office building, whilst the Rx was situated on the first floor.

#### Corridor junction scenario

**Fig. 6:**
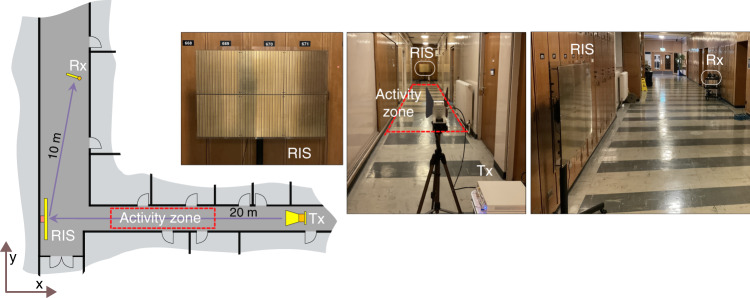
Corridor junction experiment setup. Transmitter and receiver form a virtual line of sight link via the RIS placed at the corridor junction. Dashed lines indicate the activity zone in which the experiments were performed.

The corridor junction experiment was carried out in a corridor junction, as depicted in Fig. 6, where Tx and Rx are located in adjacent corridors. The Tx consisted of a Universal Software Radio Peripheral (USRP) X300 connected to a standard gain horn antenna with 10 dB gain. The Rx consisted of a USRP X310 connected to a monopole antenna with 2 dB gain. To ensure maximum interaction with the horizontally-polarised RIS, both antennas were arranged in a horizontally polarised configuration. Laptop PCs were utilised at the Tx and Rx sides to perform signal processing on the baseband USRP signals via GNURadio Companion (GRC). These were connected to the respective USRPs via an ethernet connection.

The activities (sitting, standing, and walking) were performed between Tx and RIS, as indicated by *Activity Zone* in Fig. 6. Network equipment arranged in this manner results in a Non-LOS channel whose channel characteristics are highly dependent on the Rx position and frequency of operation^26^. If an RIS is strategically placed at the junction, such that a line of sight to the RIS is achievable for the Rx and Tx, then a reliable wireless communication link can be established by optimising the anomalous reflection characteristics of the RIS^25^. The Tx-RIS and RIS-Rx distances were 20 m and 10 m, respectively. Activity zone consists of a 5 m by 1 m area situated halfway between the Tx and RIS in a corridor of width 3.3 m.

#### Multi-floor scenario

**Fig. 7:**
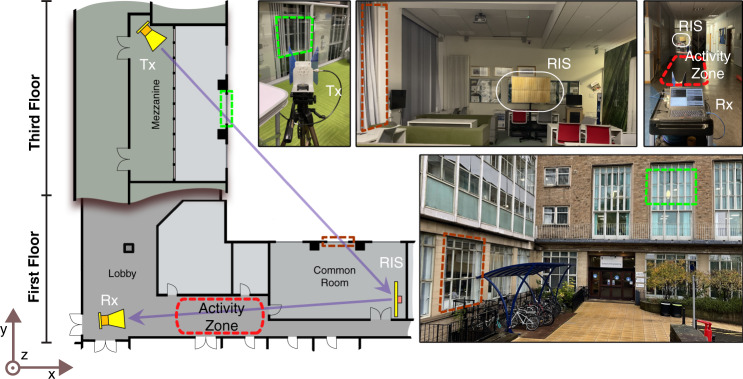
Multi-floor experiment setup. Transmitter is placed on third floor while RIS and Rx are on first floor. A LOS link is established between Tx-RIS and RIS-Rx. The activity zone is situated in a corridor section between the receiver and the RIS.

The multi-floor experiment was carried out at the University of Glasgow’s Engineering building. The Tx was placed on the third floor and the RIS and Rx were placed on the first floor as depicted in Fig. 7. Similar to the corridor junction scenario, Tx consisted of a USRP X300 connected to a standard gain horn antenna with 10 dB gain. However, due to the large channel loss, the Rx consisted of a USRP X310 connected instead to an identical horn antenna to the Tx. Both antennas were arranged in a horizontal polarisation to ensure matching with horizontally-polarised RIS.

In this scenario, activities were performed at first floor in the activity zone between RIS and Rx as indicated in Fig. 7. Firstly, a reliable communication link was established between Tx and Rx through RIS. The RIS was placed on the first floor with a LoS to the Rx. The Tx to RIS path is via two windows from a mezzanine on the 3rd floor to the area labelled *common room* on the first floor. Activities were performed between the RIS and the Rx in a 5 m by 1 m area situated 8 m from the Rx.

Each laptop is equipped with Intel(R) Core (TM) *i*7−7700 3.60 GHz processor with 16 GB RAM. The operating system was Ubuntu 16.04, which was installed as a virtual machine in each laptop. GRC was used to communicate with the USRPs on Ubuntu virtual machines, which creates flow diagrams for the USRP function. After that, the flow diagrams were turned into Python scripts, which were used to send data on Tx USRP and receive data on Rx USRP. The Tx uses orthogonal frequency division multiplexing (OFDM) to send random numbers between 0 and 255. The transmitting signal from the Tx USRP is received by the Rx after getting relayed through RIS. RIS is used to direct the beam towards Rx. The python script running on the Rx side outputs CSI as complex numbers. The amplitude values from the CSI complex numbers are then extracted from this output. Table 6 lists the system’s key configuration parameters.

**Table 6:** Configuration parameters of USRP software and hardware.

S.No	Parameters	Values
1	OFDM Subcarrier	64 carriers
2	Bit per symbol	2 bits
3	Pilot subcarrier	4
4	Devices used	USRP X300/X310
5	Channel Mapping	1 Tx, 2 Rx
5	Central Frequency	3.75 GHz
7	Data type	Int16
8	Gain(dB)	Tx 10, Rx 2

### Data collection

The data was collected for both scenarios with two participants (one male and one female) performing three different activities, sitting, standing, and walking, in experiment settings shown in Figs. 6 and 7. An empty class was introduced, which represents the reflection from the environment only in the absence of any subject in the activity zone. The ethical approval to conduct these experiments was obtained by the University of Glasgow’s Research Ethics Committee (approval no.: 300200232, 300190109).

It is important to note that the activities “Sitting” and “Standing” depict the process of conducting these activities rather than the posture or position of the individual in the sitting or standing state. Furthermore, both sitting and standing activity data included slight fluctuations in upper body, as the participants were not forced to maintain their upper bodies still and static.

For each scenario, the data were collected in two steps. In the first step, both participants performed all activities while RIS was on and then repeated the activities while RIS was off. A total of 320 data samples were collected for the corridor junction scenario, where each participant contributed equally to the data collection that is each participant collected 20 samples in each activity class for RIS-on and RIS-off. The reason to include two participants was to include maximum variation in the dataset. For similar reason, one male and one female participants were selected. Each instance of data represents the CSI data, where 1600 packets were transmitted in 4 s. That is to say that each activity was finished within 4 s and the Rx collected around 1600 CSI samples during this time.

The same data collection strategy was applied in multi-floor scenario, where a total number of 800 data samples were collected, with 50 data samples in each activity class. Similarly, each activity was performed for 4 s collecting 1600 CSI samples in each activity. The details of the collected dataset are highlighted in Table 7, where S1 represents subject 1 and S2 is subject 2. It is worth mentioning that the collected CSI values are in complex number format compromising both the amplitude and the phase information. A python script is used to extract amplitude information from those values and stored in comma-separated values (CSV) files. These CSV files are then used to train and test different machine learning algorithms after data preprocessing.

**Table 7:** Collected Dataset: number of scenarios, subjects and performed activities.

Activity	Corridor Junction				Multi-floor			
	RIS-on		RIS-off		RIS-on		RIS-off	
	S1	S2	S1	S2	S1	S2	S1	S2
Empty	20	20	20	20	50	50	50	50
Sitting	20	20	20	20	50	50	50	50
Standing	20	20	20	20	50	50	50	50
Walking	20	20	20	20	50	50	50	50

The classification of various activities is accomplished by the degree of variation in the received signal, which includes amplitude and phase variations. The received signal ’y’ at the receiver can be expressed using *y* = *Hx*, where *H* is the channel matrix and *x* is the transmitted signal. The channel H can be further expressed as^27^,

2 $$H={h}_{d}+{h}_{1}{{\Phi }}{h}_{2}^{T}$$

where *h*_*d*_ is the channel from the transmitter to receiver, *h*_1_ is the channel from the transmitter to RIS, *h*_2_ is the channel from RIS to the receiver and *T* represents the transpose. Furthermore, Φ is a tuning matrix containing the values of phase and amplitude coefficients of individual RIS elements. As the activity monitoring in the proposed work is performed using an Non-LOS scenario eliminating the direct path *h*_*d*_, Eq. (2) could be summarised as follows:

3 $$H={h}_{1}{{\Phi }}{h}_{2}^{T}$$

**Fig. 8:**
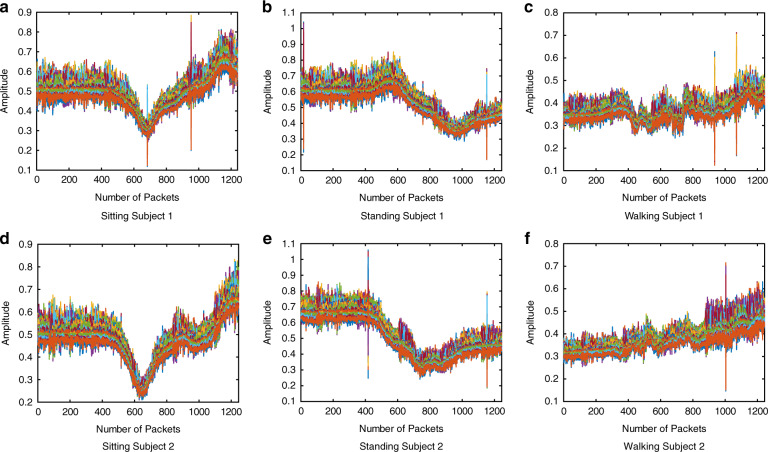
CSI data samples of activities with RIS-on in corridor junction scenario. **a** Subject 1 in sitting posture. **b** Subject 1 in standing posture. **c** Subject 1 in walking posture. **d** Subject 2 in sitting posture. **e** Subject 2 in standing posture. **f** Subject 2 in walking posture.

**Fig. 9:**
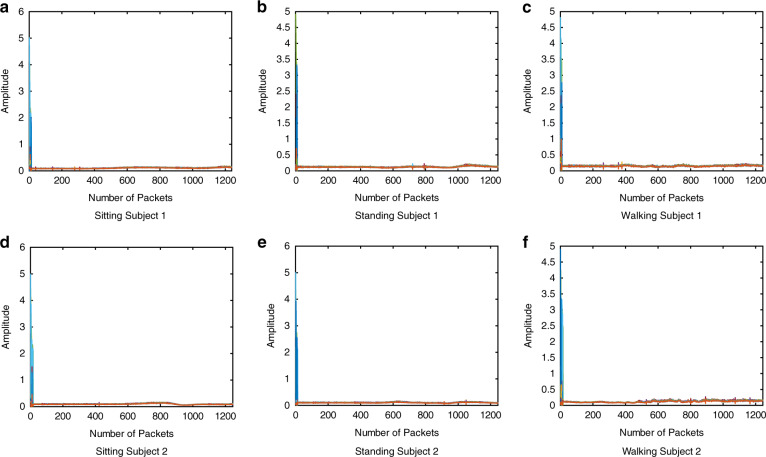
CSI data samples of activities with RIS-off in corridor junction scenario. **a** Subject 1 in sitting posture. **b** Subject 1 in standing posture. **c** Subject 1 in walking posture. **d** Subject 2 in sitting posture. **e** Subject 2 in standing posture. **f** Subject 2 in walking posture.

In this work, we considered the signal amplitudes only, which are presented in Figs. 8 and 9 for RIS-on and RIS-off, respectively, represent the CSI patterns (amplitude) of different body movements, i.e. sitting, standing, and walking in the corridor junction scenario. Different colours in each figure represents the 64 subcarriers of the OFDM signal. *Y*-axis of each sub-figure represents the amplitude of the subcarriers while number of received packets are displayed on *x*-axis. Note that the received signal amplitude in the case of RIS-off is too low to correctly distinguish different activities. On the other hand, turning on RIS gives significant changes in the received signal patterns, which are unique for different activities but similar for different subjects. A clear resemblance between sitting, standing, and walking activities of S1 and S2 can be observed in Fig. 8, which encourages the generalisation of the proposed scheme to a broad range of users.

### Data preprocessing

**Fig. 10:**
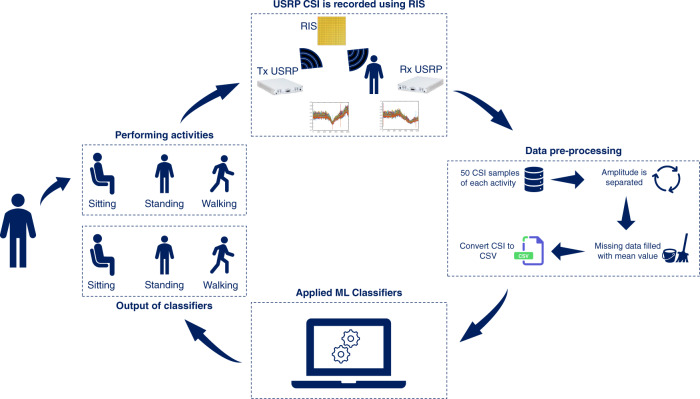
Data flow diagram of RIS-enabled human activity monitoring system.

Once the data are collected and stored in CSV files, it is not unusual that it has some missing data due to loss of received packets, which requires data equalisation. We use *Scikit*, a commonly used data analysis toolkit in Python^28^, for data preprocessing and applying machine learning algorithms. Furthermore, *Pandas*, a python package, is used to interpret CSV files. It transforms CSV files into python dataframes, which are subsequently analysed with *SciKit*^29^. The labels are added in the first column of dataframes. Due to data length miss match, not a number (NaN) values are produced in the dataset obtained by merging the data frames of each sample. These NaN values are replaced with the mean of each row by using a built in function of *SciKit*, called *SimpleImputer*. It is worth mentioning that this kind of data equalisation did not affect the overall pattern of the data. This data after equalisation was fed to different machine learning algorithms, namely RF, ET and MLP. These algorithms were chosen after extensive study of various machine learning algorithm on the dataset. The data flow diagram of the whole process is shown in Fig. 10.

### Machine learning

The proposed IWW-enabled human activity monitoring system is evaluated using three different machine learning algorithms. The evaluation parameter considered in our experiment is the accuracy of successfully identifying different body movements. The accuracy of each algorithm was evaluated on individual participants’ dataset separately and on the combined dataset. In order to have a thorough investigation, the accuracy was measured into two different ways, (i) *k*-fold cross validation and (ii) test-train split. *k*-fold cross-validation is a popular method for evaluating the performance of a machine learning algorithm, where *k* refers to the number of groups that a given data sample is to be split into. In our experiments, we used a variant of *k*-fold cross validation that is repeated stratified (RS) *k*-fold cross-validation, using *scikit*-learn python ML library’s *RepeatedStratifiedKFold* class. In particular, we consider (3) 10-fold cross validation, which means that the repetition cycle is selected as 3 and cross validation as 10.

The other evaluation approach considered in this work is test-train split that makes predictions based on data that has not been used to train the model. This approach divides the dataset into two parts. The training dataset is the first part of data to which the machine learning model is applied. The test dataset is the second part of the dataset that is used to evaluate the performance. In this work, 80% of the data is used for training and 20% for testing. The parameters used to train the classifiers are listed in Table 8.

**Table 8:** Parameters of machine learning algorithms.

S.No	Algorithm	Hyperparameters
1	Random Forest	n-estimators : 20, max-features: [auto, log2], max-depth: range(2,20), criterion: gini
2	Extra Tree	n-estimators : 20, max-features: range(1, 21), min-samples_*s*_*plit*: range(2, 15)
3	Multilyer Perceptron	activation: [relu, softmax], epochs=150, batch-size=32, verbose=2, optimizer: adam

### System generalisation

The activity monitoring problem can be classified into two types: macro activities and micro activities, where macro activities contain significant movements resulting in distinct variations in the received signal. This includes sitting, standing, walking, running and any other day-to-day activities. On the other hand, the micro activities induce variation on an exceedingly small scale and the proposed model would require some changes such as using mm-wave frequency to obtain higher resolution, which can be obtained by scalability of current system. Micro activities might include sign language with various finger movements, lip movements, etc. The existing model is based on the classification of macro activities and is not tailored to be used for micro activities monitoring. Further, the daily-life macro-activities, where the whole body is participating in the activity, are generally limited in number (sitting, standing, running, walking, fall, pick up item, etc.) and are commonly distinct. Hence the proposed system can be easily generalised to include any macro-movements.

Further, the problem of muti-person activity monitoring with RF sensing has been addressed in one of our previous works^3^, which was the first 5G-enabled multi-person activity monitoring system with 4 subjects in 16 unique activity combinations. The proposed system combined the subject count and activities performed in different classes together, resulting in simultaneous identification of the occupancy count and activities performed. The proposed system^3^ worked in the LOS environment. However, the generalisation of such a system in the Non-LOS environment using RIS is possible, and is left for the future work.
